# Supplementary material for: Global distribution of research efforts, disease burden, and impact of US public funding withdrawal
Source: Nat Med. 2025 Aug 27;31(9):3101–9. doi: 10.1038/s41591-025-03923-0 (PMC12443587; doi:10.1038/s41591-025-03923-0)
Supplement: Supplementary file 1 — Supplementary Figs. 1–12. [file 41591_2025_3923_MOESM1_ESM.pdf]

nature medicine

Supplementary information https://doi.org/10.1038/s41591-025-03923-0

Global distribution of research efforts, disease burden, and impact of US public funding withdrawal

In the format provided by the authors and unedited

Supplementary Information

Global distribution of research efforts, disease burden, and impact of US public funding withdrawal

Leo Schmallenbach, Maximilian Bley, Till W. Bärnighausen, Cassidy R. Sugimoto, Carolin Lerchenmüller, Marc J. Lerchenmueller

Table of contents

Supplementary Figure 1

- Supplementary Figure 1A. Research-Disease Divergence measured by Population Stability Index
- Supplementary Figure 1B. Research-Disease Divergence measured by Hellinger Distance
- Supplementary Figure 1C. Research-Disease Divergence measured by Jensen-Shannon Divergence

Supplementary Figure 2

- Supplementary Figure 2A. Contributions of research and disease burden proxied by mortality to changes in divergence
- Supplementary Figure 2B. Contributions of research and disease burden proxied by prevalence to changes in divergence

Supplementary Figure 3

- Distribution of DALYs and research across diseases by year and geographic region

Supplementary Figure 4

- Rank changes of DALYs and research by disease from 2019 relative to 1999

Supplementary Figure 5

- Supplementary Figure 5A. Differentiating research that acknowledges funding versus not
- Supplementary Figure 5B. Differentiating research with industry involvement and Phase 3 clinical trials with industry sponsors
- Supplementary Figure 5C. Differentiating research related to public health, health system, and clinical applications

Supplementary Figure 6

- Statistics for geo-locating papers based on first authors versus all authors

Supplementary Figure 7

- Supplementary Figure 7A. Projected distribution of DALYs across diseases by year
- Supplementary Figure 7B. Projected distribution of research across diseases by year
- Supplementary Figure 7C. Projected distribution of research exclusive of international research with public U.S. funding across diseases by year

Supplementary Figure 8

- Research-Disease Divergence for level 3 disease causes

Supplementary Figure 9

- Supplementary Figure 9A. Distribution of DALYs and research for level 3 disease causes related to level 2 cause cardiovascular diseases
- Supplementary Figure 9B. Distribution of DALYs and research for level 3 disease causes related to level 2 cause neoplasms

Supplementary Figure 10

- LLM custom prompt

Supplementary Figure 11

- Triangulated performance metrics

Supplementary Figure 12

- LLM versus ICD recall analysis

Supplementary Figure 1

Supplementary Figure 1A. Research-Disease Divergence measured by Population Stability Index

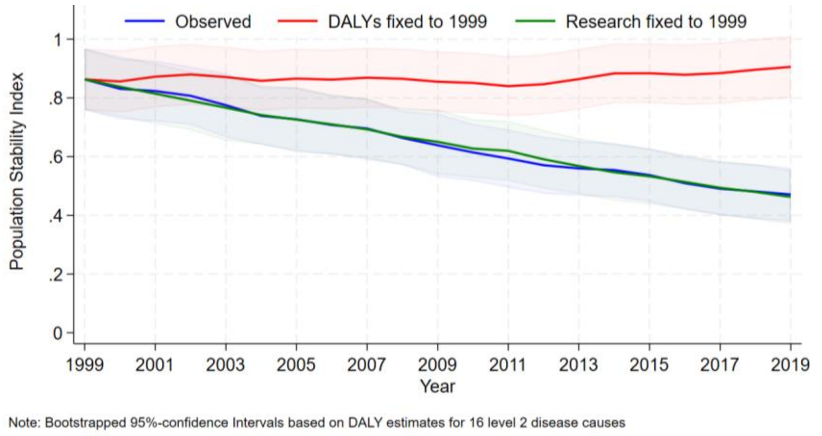

Supplementary Figure 1B. Research-Disease Divergence measured by Hellinger Distance

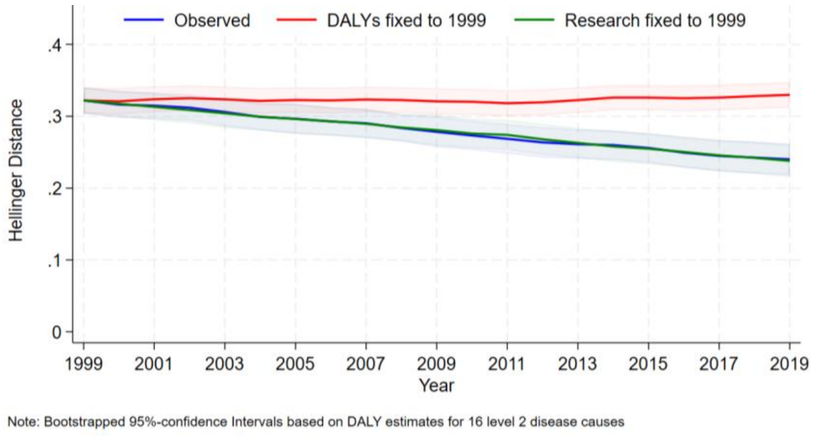

Supplementary Figure 1C. Research-Disease Divergence measured by Jensen-Shannon Divergence

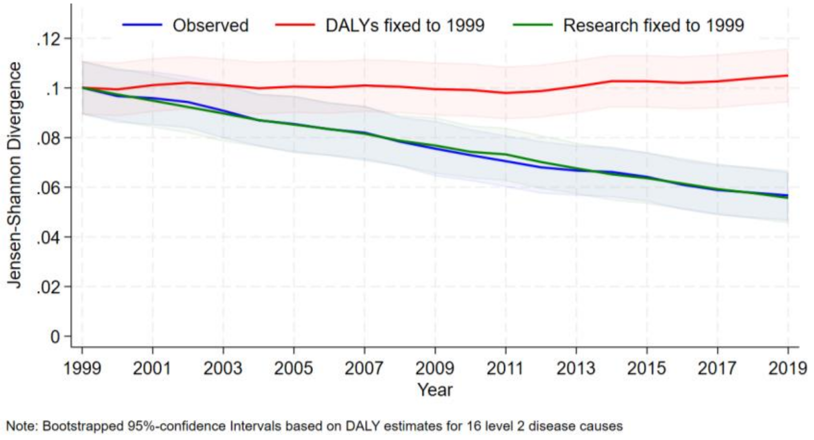

Supplementary Figure 2

Supplementary Figure 2A. Contributions of research and disease burden proxied by mortality to changes in divergence

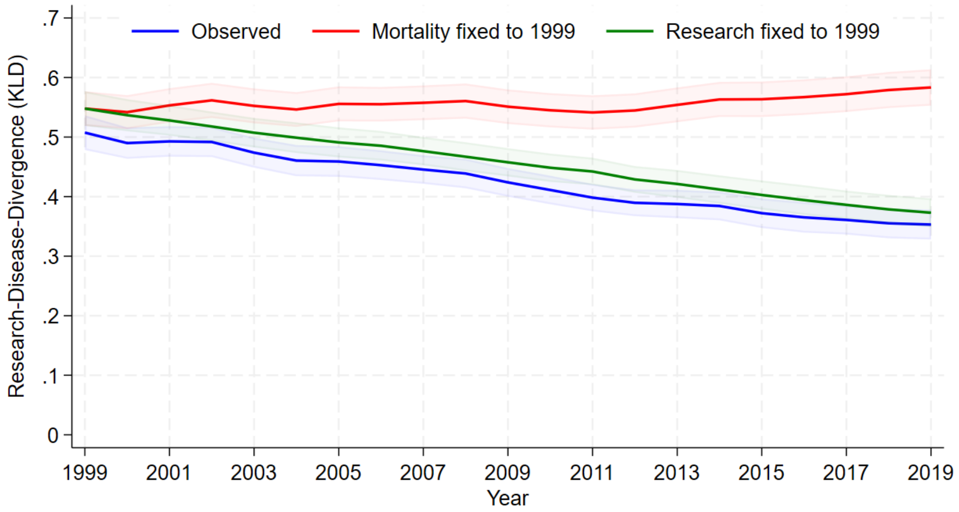

Note: Bootstrapped 95%-confidence Intervals based on Mortality estimates for 15 level 2 disease causes

Supplementary Figure 2B. Contributions of research and disease burden proxied by prevalence to changes in divergence

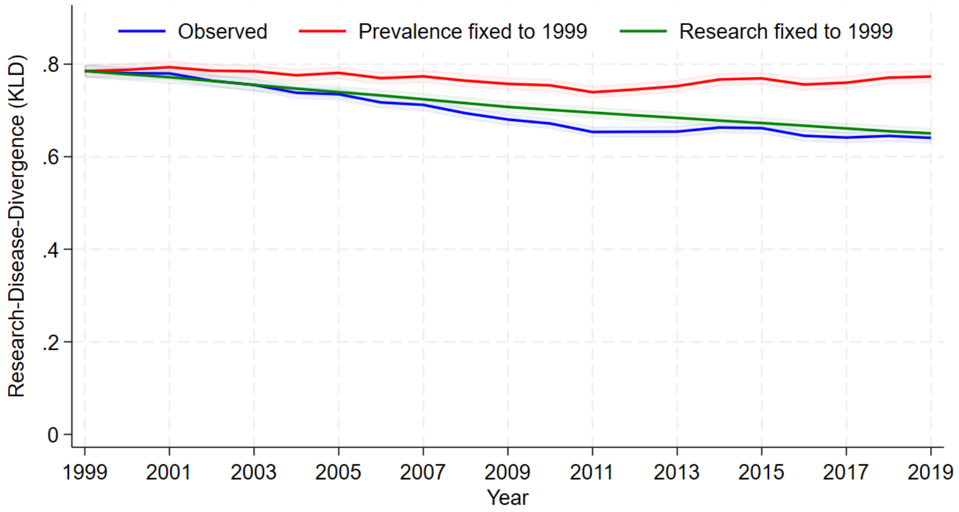

Note: Bootstrapped 95%-confidence Intervals based on Prevalence estimates for 16 level 2 disease causes

Supplementary Figure 3

Distribution of DALYs and research across diseases by year and region

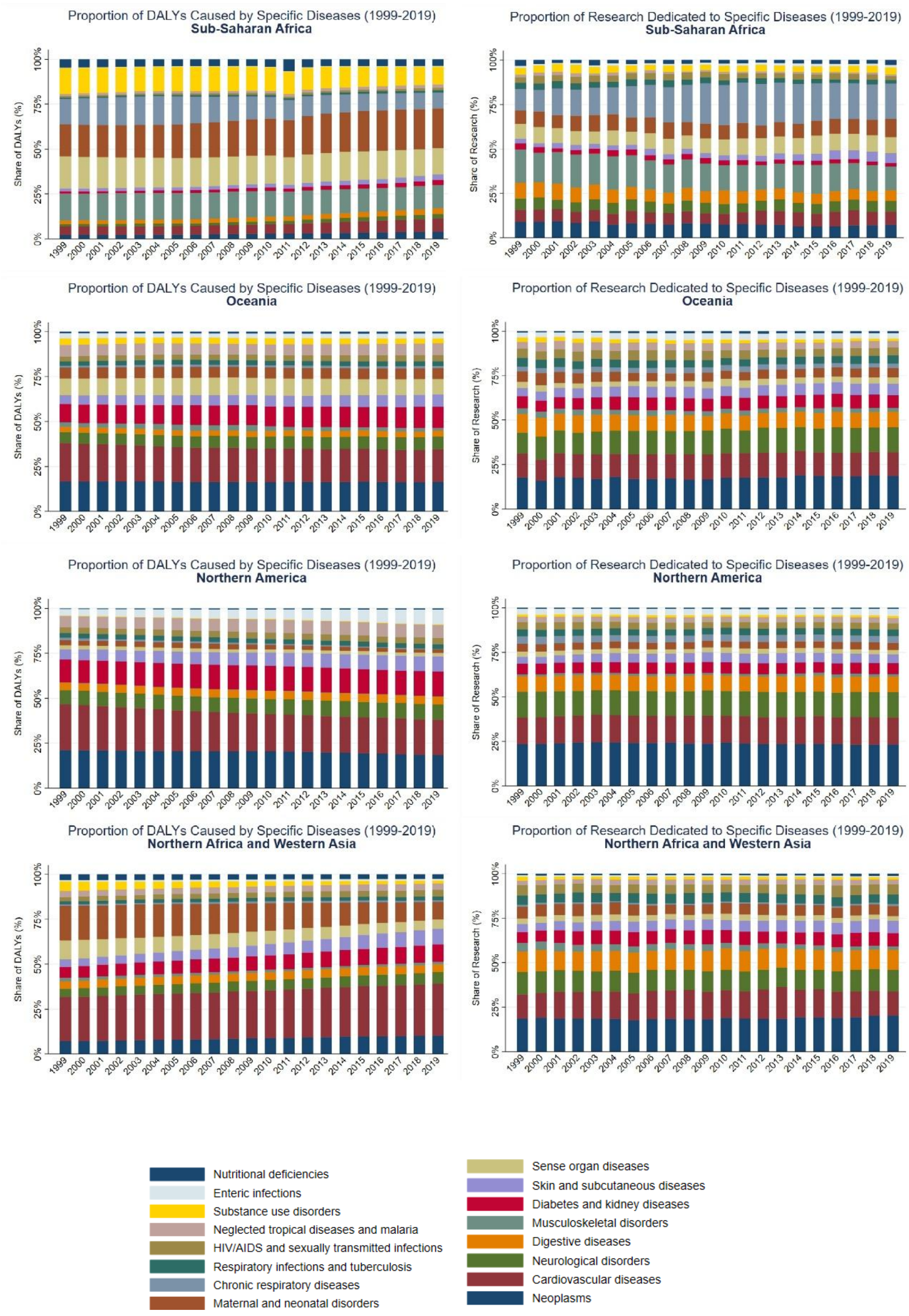

Supplementary Figure 3

Distribution of DALYs and research across diseases by year and region (continued)

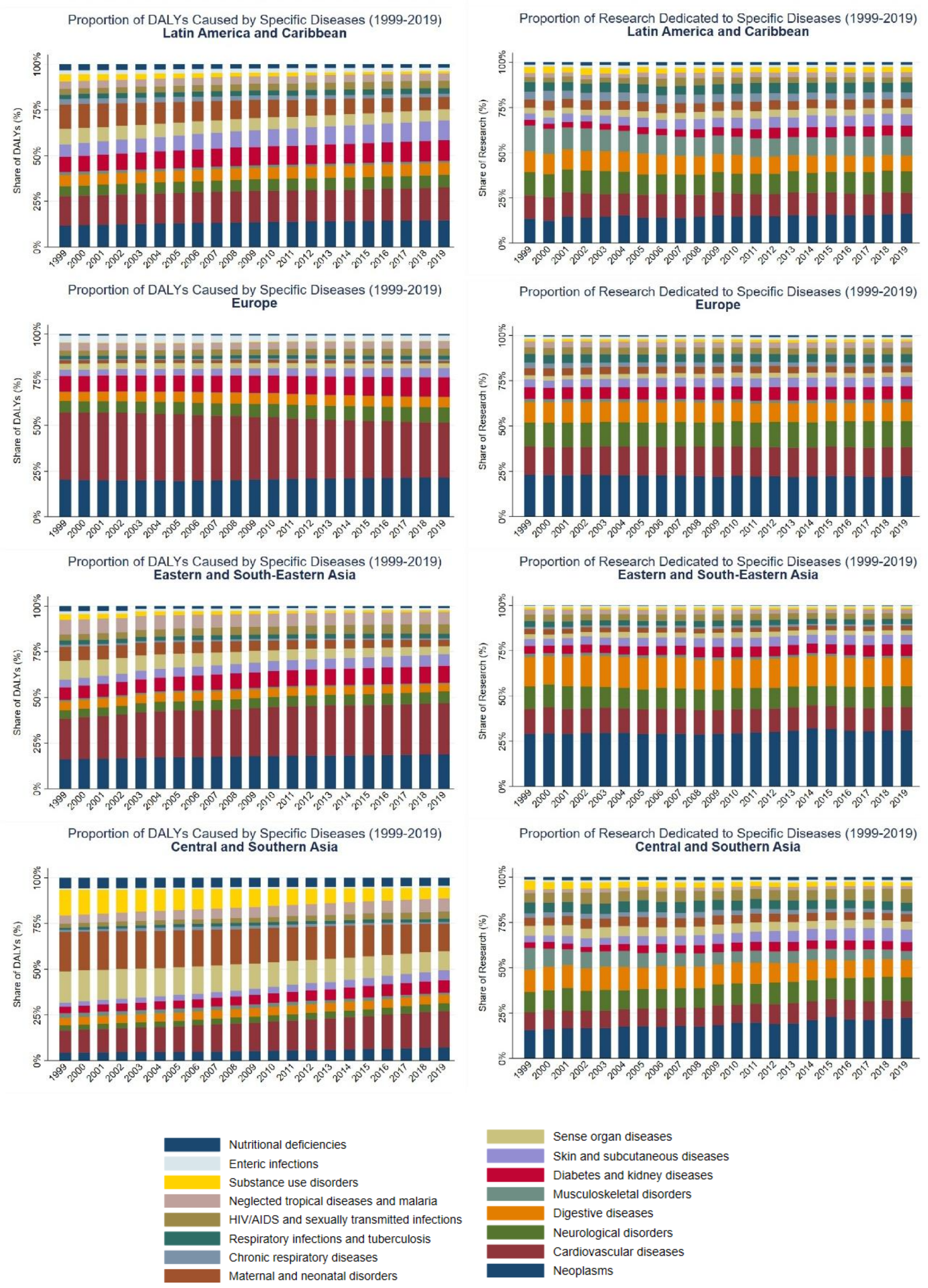

Supplementary Figure 4

Rank changes of DALYs and research by disease from 2019 relative to 1999

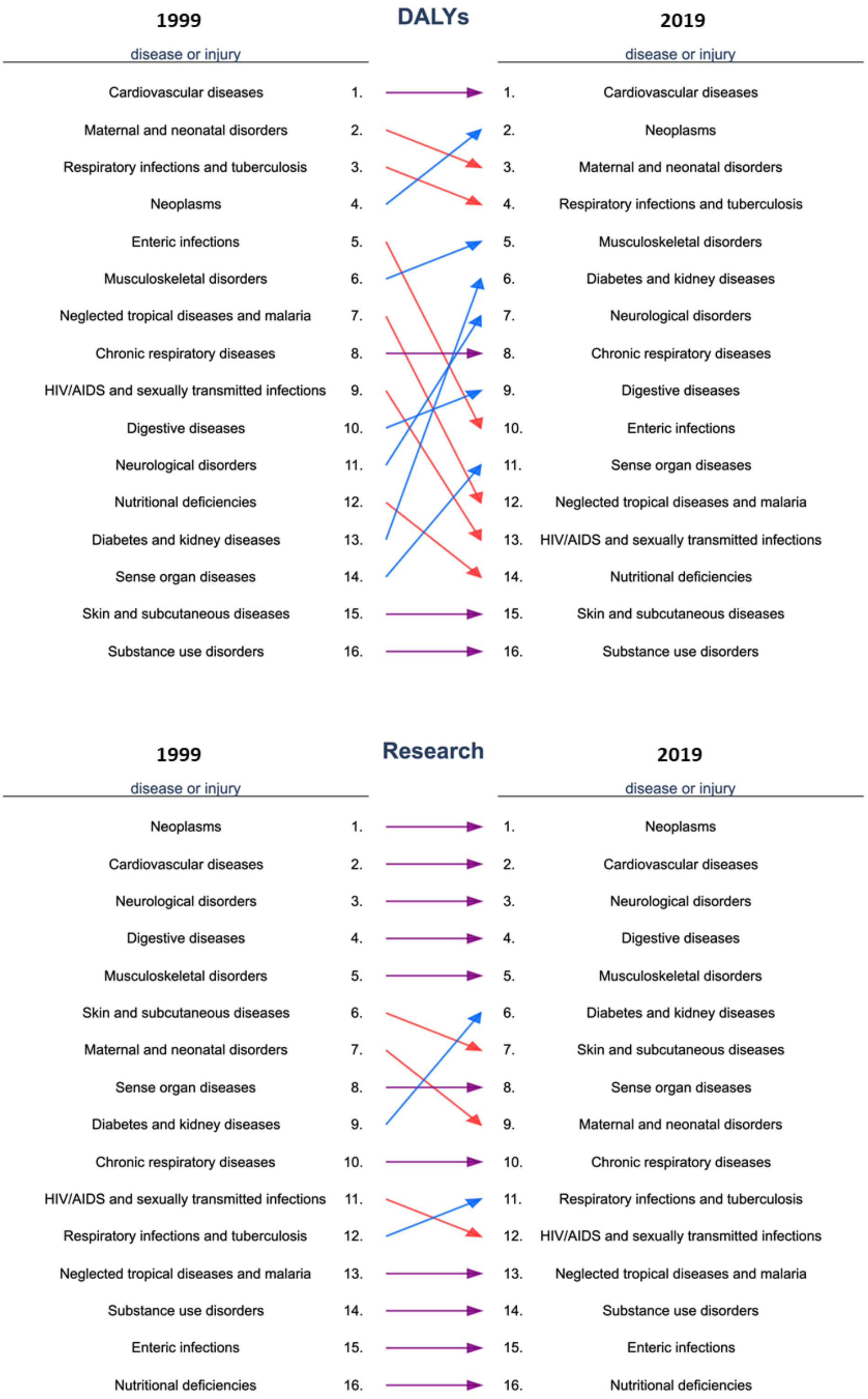

Supplementary Figure 5

Supplementary Figure 5A. Differentiating research that acknowledges funding versus not

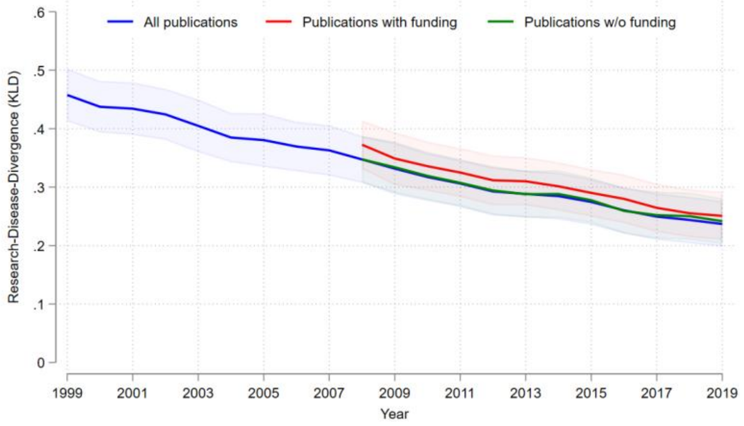

Note: Bootstrapped 95%-confidence Intervals based on DALY estimates for 16 level 2 disease causes.
Funding data available as of 2008.

Supplementary Figure 5B. Differentiating research with industry involvement and Phase 3 clinical trials with industry sponsors

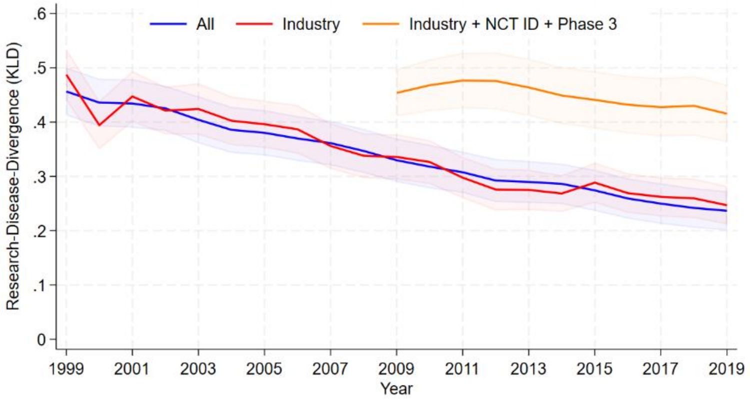

Note: Bootstrapped 95%-confidence Intervals based on DALY estimates for 16 level 2 disease causes.

Supplementary Figure 5C. Differentiating research related to public health, health system, and clinical applications

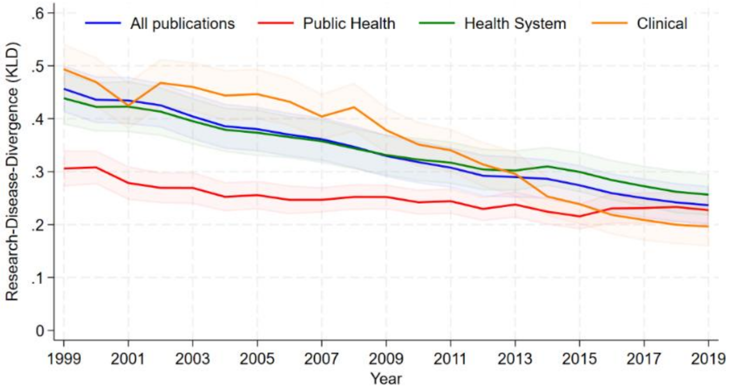

Note: Bootstrapped 95%-confidence Intervals based on DALY estimates for 16 level 2 disease causes.

Supplementary Figure 6

Statistics for geo-locating papers based on first authors versus all authors

Region	% DALYs	% Any Authorship	% First Authorship
Central and Southern Asia	26%	4%	4%
Eastern and South-Eastern Asia	25%	23%	26%
Sub-Saharan Africa	21%	2%	1%
Europe	10%	30%	31%
Latin America and Caribbean	7%	4%	4%
Northern Africa and Western Asia	5%	4%	4%
Northern America	5%	29%	28%
Oceania	0%	4%	3%

Supplementary Figure 7

<u>Supplementary Figure 7A.</u> Projected distribution of DALYs across diseases by year.

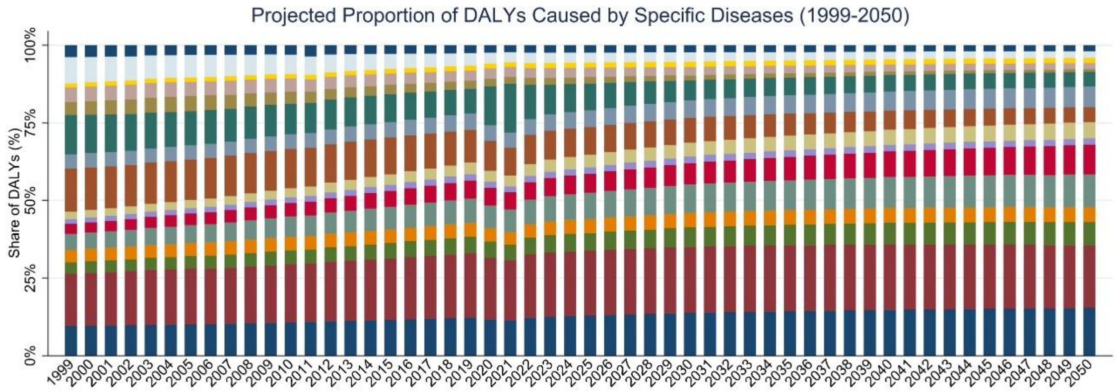

<u>Supplementary Figure 7B.</u> Projected distribution of research across diseases by year.

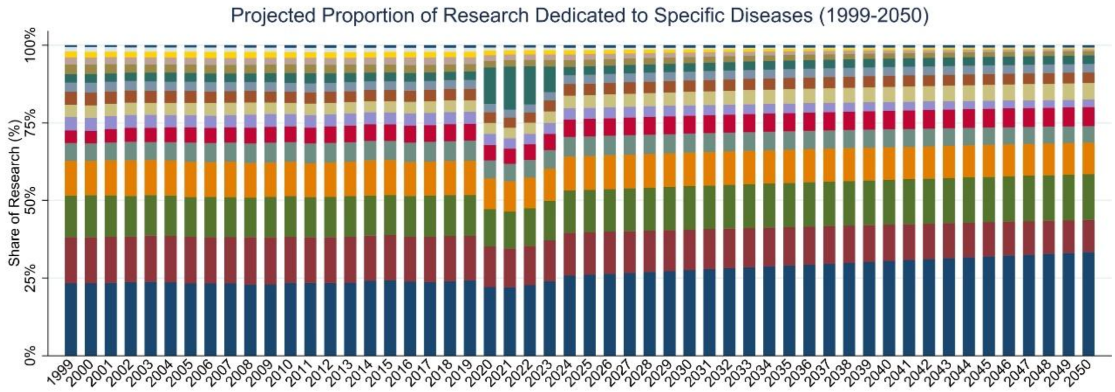

<u>Supplementary Figure 7C.</u> Projected distribution of research exclusive of international research with public U.S. funding across diseases by year

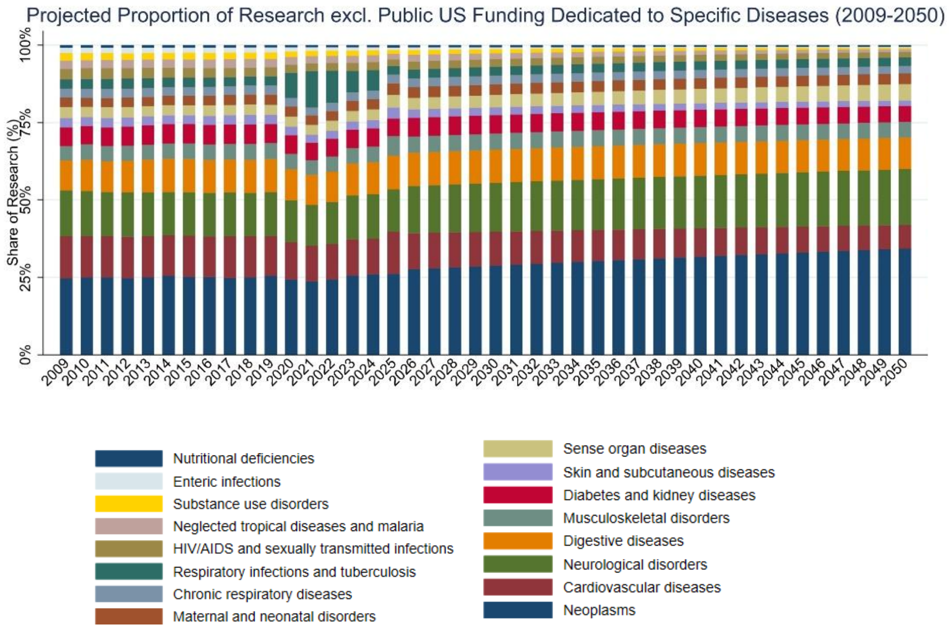

Supplementary Figure 8

Research-Disease Divergence for level 3 disease causes

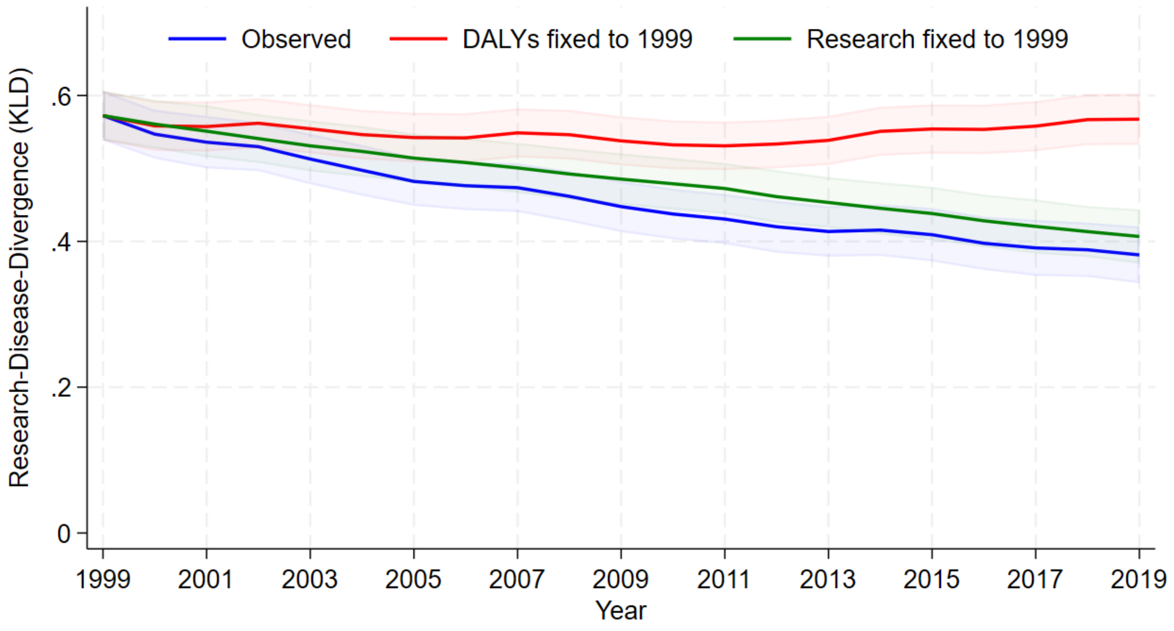

Note: Bootstrapped 95%-confidence Intervals based on DALY estimates for 115 level 3 disease causes

Supplementary Figure 9

Supplementary Figure 9A. Distribution of DALYs and research for level 3 disease causes related to Level 2 cause cardiovascular diseases.

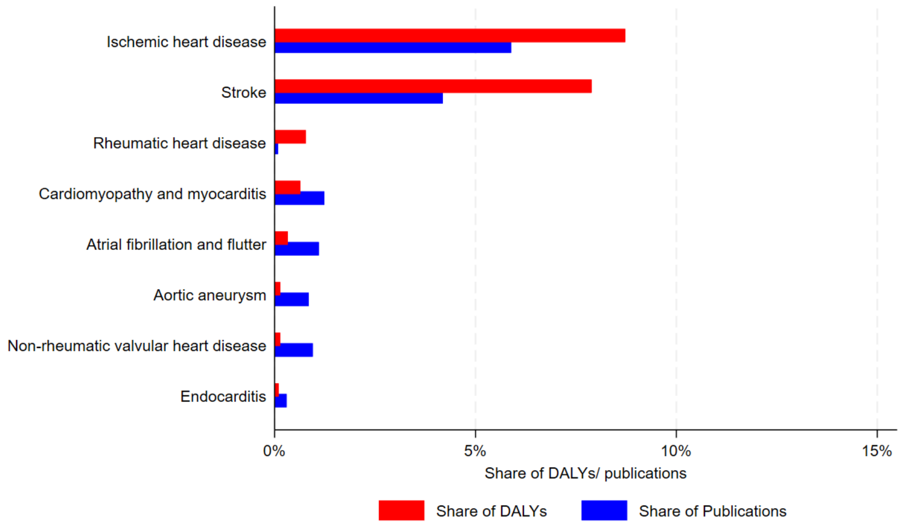

Supplementary Figure 9B. Distribution of DALYs and research for level 3 disease causes related to level 2 cause neoplasms

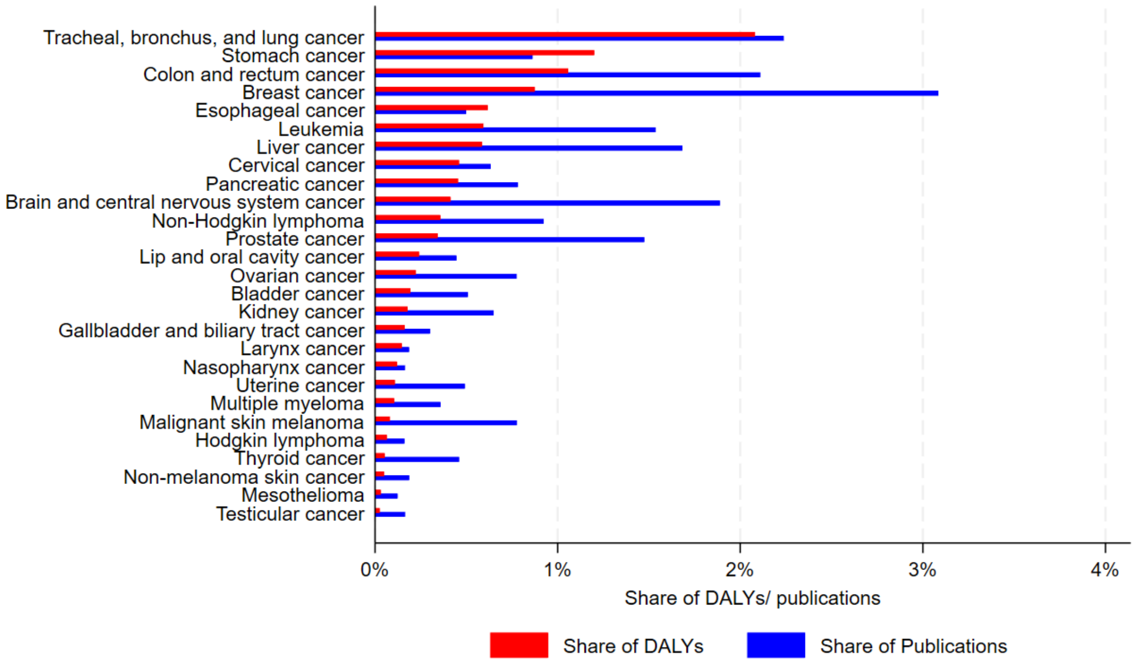

Supplementary Figure 10

LLM custom prompt

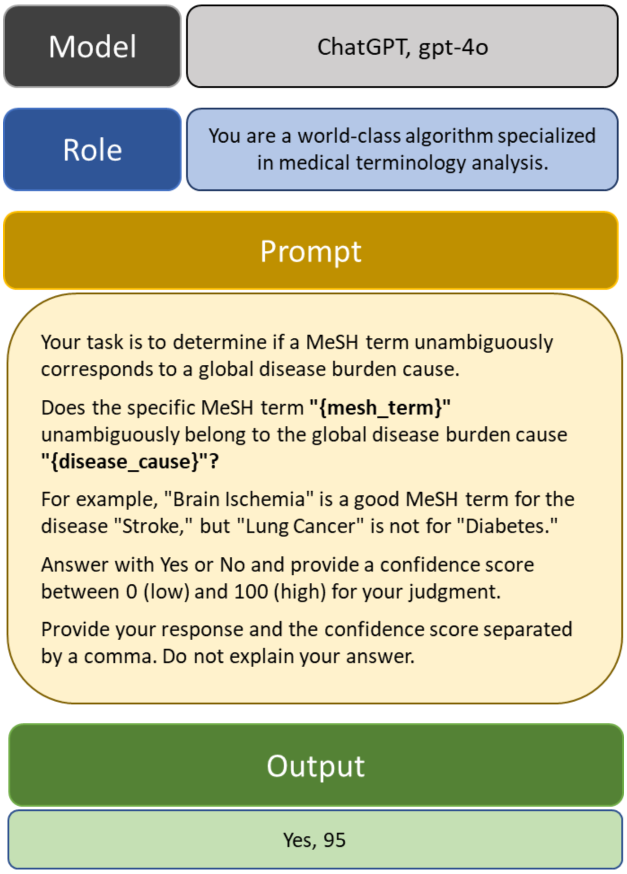

Supplementary Figure 11

Triangulated performance metrics

Evaluation ChatGPT vs. ICD-10 - Cardiovascular Diseases - at the Paper Level, Publications 1999-2021

ChatGPT

		Annotated	Not Annotated	
**Expert**	Annotated	1,324,710	34,589	**1,359,299**
	Not Annotated	46,216	182,625	**228,841**
		**1,370,926**	**217,214**	**1,588,140**

ICD-10

		Annotated	Not Annotated	
**Expert**	Annotated	867,638	491,661	**1,359,299**
	Not Annotated	32,735	196,106	**228,841**
		**900,373**	**687,767**	**1,588,140**

**Compared to Experts**	**ChatGPT**	**ICD-10**
Accuracy	94.9%	67.0%
Precision	96.6%	96.4%
Recall	97.5%	63.8%

Supplementary Figure 12
LLM versus ICD recall analysis

		ChatGPT		ICD	
**Journal Cause**	**# articles**	**# articles with same cause**	**% articles with same cause**	**# articles with same cause**	**% articles with same cause**
Cardiovascular diseases	5053	4843	95.84%	3482	68.91%
Chronic respiratory diseases	1323	1306	98.72%	1278	96.60%
Diabetes and kidney diseases	4645	4233	91.13%	3538	76.17%
Digestive diseases	1194	1124	94.14%	670	56.11%
HIV/AIDS and sexually transmitted infections	8793	8319	94.61%	7680	87.34%
Musculoskeletal disorders	7485	6343	84.74%	4147	55.40%
Neglected tropical diseases and malaria	5876	5738	97.65%	5742	97.72%
Neoplasms	10294	10092	98.04%	4429	43.03%
Neurological disorders	19754	19043	96.40%	15873	80.35%
**Total**	**64417**	**61041**	**94.76%**	**46839**	**72.71%**
