# Supplementary material for: Global distribution of research efforts, disease burden, and impact of US public funding withdrawal
Source: Nat Med. 2025 Aug 27;31(9):3101–9. doi: 10.1038/s41591-025-03923-0 (PMC12443587; doi:10.1038/s41591-025-03923-0)
Supplement: Supplementary file 2 — Reporting Summary [file 41591_2025_3923_MOESM2_ESM.pdf]

nature portfolio

Corresponding author(s): Marc Lerchenmüller

Last updated by author(s): Jul 14, 2025

Reporting Summary

Nature Portfolio wishes to improve the reproducibility of the work that we publish. This form provides structure for consistency and transparency in reporting. For further information on Nature Portfolio policies, see our Editorial Policies and the Editorial Policy Checklist.

Statistics

For all statistical analyses, confirm that the following items are present in the figure legend, table legend, main text, or Methods section.

n/a	Confirmed	
☐	☒	The exact sample size (n) for each experimental group/condition, given as a discrete number and unit of measurement
☒	☐	A statement on whether measurements were taken from distinct samples or whether the same sample was measured repeatedly
☒	☐	The statistical test(s) used AND whether they are one- or two-sided<br>*Only common tests should be described solely by name; describe more complex techniques in the Methods section.*
☐	☒	A description of all covariates tested
☒	☐	A description of any assumptions or corrections, such as tests of normality and adjustment for multiple comparisons
☐	☒	A full description of the statistical parameters including central tendency (e.g. means) or other basic estimates (e.g. regression coefficient) AND variation (e.g. standard deviation) or associated estimates of uncertainty (e.g. confidence intervals)
☒	☐	For null hypothesis testing, the test statistic (e.g. F, t, r) with confidence intervals, effect sizes, degrees of freedom and P value noted<br>*Give P values as exact values whenever suitable.*
☒	☐	For Bayesian analysis, information on the choice of priors and Markov chain Monte Carlo settings
☒	☐	For hierarchical and complex designs, identification of the appropriate level for tests and full reporting of outcomes
☒	☐	Estimates of effect sizes (e.g. Cohen's d, Pearson's r), indicating how they were calculated

Our web collection on statistics for biologists contains articles on many of the points above.

Software and code

Policy information about availability of computer code

Data collection	No software was used to collect data.
Data analysis	All analyses were performed in Stata 18.

For manuscripts utilizing custom algorithms or software that are central to the research but not yet described in published literature, software must be made available to editors and reviewers. We strongly encourage code deposition in a community repository (e.g. GitHub). See the Nature Portfolio guidelines for submitting code & software for further information.

Data

Policy information about availability of data

All manuscripts must include a data availability statement. This statement should provide the following information, where applicable:

- Accession codes, unique identifiers, or web links for publicly available datasets
- A description of any restrictions on data availability
- For clinical datasets or third party data, please ensure that the statement adheres to our policy

We collected data from PubMed via the PubMed XML bulk download, Web of Science (WoS), iCite from the NIH Collection, and the Global Burden of Disease database from healthdata.org.The data assembled in this study will be deposited in the Figshare database.

Research involving human participants, their data, or biological material

Policy information about studies with human participants or human data. See also policy information about sex, gender (identity/presentation), and sexual orientation and race, ethnicity and racism.

Reporting on sex and gender	N/A
Reporting on race, ethnicity, or other socially relevant groupings	N/A
Population characteristics	N/A
Recruitment	N/A
Ethics oversight	N/A

Note that full information on the approval of the study protocol must also be provided in the manuscript.

Field-specific reporting

Please select the one below that is the best fit for your research. If you are not sure, read the appropriate sections before making your selection.

☐ Life sciences ☒ Behavioural & social sciences ☐ Ecological, evolutionary & environmental sciences

For a reference copy of the document with all sections, see nature.com/documents/nr-reporting-summary-flat.pdf

Behavioural & social sciences study design

All studies must disclose on these points even when the disclosure is negative.

Study description	This study uses a quantitative design to develop a novel dataset linking global disease burden data with disease-related scientific publications. This enables a longitudinal analysis of the alignment between health needs and research activity. The resulting data foundation supports evidence-based agenda setting across different levels of disease specificity and geographic context.
Research sample	This study draws on existing datasets from two primary sources: (1) the Global Burden of Disease (GBD) database, which provides level 2 disease categories and corresponding Disability-Adjusted Life Years (DALYs) for all ages and both sexes from 1999 to 2021 across 204 countries and territories, and (2) PubMed, which indexes biomedical research articles annotated with Medical Subject Headings (MeSH). Using a large language model (LLM)-based approach, we linked MeSH terms to GBD disease categories, resulting in 9.7 million article–disease cause connections. The sample includes the global scientific literature and population-level disease burden data, making it representative at the country and disease levels. This approach enables a comprehensive assessment of how research outputs align with health needs over time and across regions.
Sampling strategy	No sampling procedure or sample size calculation was performed, as the study used the full population of disease-related publications in PubMed and complete GBD data. This comprehensive approach ensures sufficient coverage for robust, large-scale analysis.
Data collection	The data collection procedure relied exclusively on publicly available secondary datasets and did not involve direct interaction with human participants. We extracted disease-related publications from PubMed, using Medical Subject Headings (MeSH) from the C-branch to identify articles relevant to specific disease causes. These MeSH terms were then linked to level 2 causes in the Global Burden of Disease (GBD) database using a custom-developed large language model (LLM)–based classification tool. This tool systematically evaluated the semantic fit between each MeSH term and GBD cause to establish article–disease links. No additional individuals were present during data collection, and researcher blinding was not applicable, as the study design was purely computational and did not involve experimental conditions or human subjects.
Timing	Data was collected in July 2024.
Data exclusions	No data were excluded from the analyses. All available disease-related publications with geo-located first authors in PubMed and corresponding GBD data were included.
Non-participation	No participants were involved in this study; it is based entirely on secondary analysis of publicly available datasets.
Randomization	Please state how many participants dropped out/declined participation and the reason(s) given OR provide response rate OR state that no participants dropped out/declined participation OR if no participants were involved in the study, please state here accordingly

Reporting for specific materials, systems and methods

We require information from authors about some types of materials, experimental systems and methods used in many studies. Here, indicate whether each material, system or method listed is relevant to your study. If you are not sure if a list item applies to your research, read the appropriate section before selecting a response.

Materials & experimental systems

n/a	Involved in the study
☒	☐ Antibodies
☒	☐ Eukaryotic cell lines
☒	☐ Palaeontology and archaeology
☒	☐ Animals and other organisms
☒	☐ Clinical data
☒	☐ Dual use research of concern
☒	☐ Plants

Methods

n/a	Involved in the study
☒	☐ ChIP-seq
☒	☐ Flow cytometry
☒	☐ MRI-based neuroimaging

Plants

Seed stocks	*Report on the source of all seed stocks or other plant material used. If applicable, state the seed stock centre and catalogue number. If plant specimens were collected from the field, describe the collection location, date and sampling procedures.*
Novel plant genotypes	*Describe the methods by which all novel plant genotypes were produced. This includes those generated by transgenic approaches, gene editing, chemical/radiation-based mutagenesis and hybridization. For transgenic lines, describe the transformation method, the number of independent lines analyzed and the generation upon which experiments were performed. For gene-edited lines, describe the editor used, the endogenous sequence targeted for editing, the targeting guide RNA sequence (if applicable) and how the editor was applied.*
Authentication	*Describe any authentication procedures for each seed stock used or novel genotype generated. Describe any experiments used to assess the effect of a mutation and, where applicable, how potential secondary effects (e.g. second site T-DNA insertions, mosiacism, off-target gene editing) were examined.*
